# Decrease in Dengue virus-2 infection and reduction of cytokine/chemokine production by *Uncaria guianensis* in human hepatocyte cell line Huh-7

**DOI:** 10.1590/0074-02760160323

**Published:** 2017-06

**Authors:** Cíntia da Silva Mello, Ligia Maria Marino Valente, Thiago Wolff, Raimundo Sousa Lima-Junior, Luciana Gomes Fialho, Cintia Ferreira Marinho, Elzinandes Leal Azeredo, Luzia Maria Oliveira-Pinto, Rita de Cássia Alves Pereira, Antonio Carlos Siani, Claire Fernandes Kubelka

**Affiliations:** 1 Fundação Oswaldo Cruz-Fiocruz, Instituto Oswaldo Cruz, Laboratório de Imunologia Viral, Rio de Janeiro, RJ, Brasil; 2 Universidade Federal do Rio de Janeiro, Instituto de Química, Rio de Janeiro, RJ, Brasil; 3 Universidade do Estado do Amazonas, Escola Normal Superior, Manaus, AM, Brasil; 4 Embrapa Agroindústria Tropical, Fortaleza, CE, Brasil; 5 Fundação Oswaldo Cruz-Fiocruz, Instituto de Tecnologia em Fármacos, Rio de Janeiro, RJ, Brasil

**Keywords:** dengue, Uncaria guianensis, traditional medicine, immunomodulation, antivirals, kaempferitrin

## Abstract

**BACKGROUND:**

Dengue fever may present hemorrhages and cavitary effusions as result of exacerbated immune responses. We investigated hydro-alcoholic extracts from leaves (UGL) and bark (UGB) of the medicinal species *Uncaria guinanensis* with respect to antiviral effects in Dengue virus (DENV) infection and in immunological parameters associated with *in vivo* physiopathological features.

**METHODS:**

Chemical profiles from UGB or UGL were compared in thin layer chromatography and ^1^H nuclear magnetic resonance using flavonoid compounds and a pentacyclic oxindole alkaloid-enriched fraction as references. DENV-2-infected hepatocytes (Huh-7) were treated with extracts. Cell viability, DENV antigens and immunological factors were detected by enzyme-linked immunosorbent assay (ELISA) or flow cytometry.

**FINDINGS:**

The UGL mainly differed from UGB by selectively containing the flavonoid kaempferitrin. UGB and UGL improved hepatocyte viability. Both extracts reduced intracellular viral antigen and inhibited the secretion of viral non-structural protein (NS1), which is indicative of viral replication. Reduction in secretion of macrophage migration inhibitory factor was achieved by UGB, of interleukin-6 by UGL, and of interleukin-8 by both UGB and UGL. MAIN

**CONCLUSIONS:**

The *U. guianensis* extracts presented, antiviral and immunomodulatory effects for DENV and possibly a hepatocyte-protective activity. Further studies may be performed to consider these products as potential candidates for the development of an herbal product for the future treatment of dengue.

Dengue virus (DENV) belongs to the genus *Flavivirus*, family *Flaviviridae* and is an enveloped positive sense RNA virus consisting in four closely-related but antigenically distinct serotypes known to co-circulate (DENV1-4) (WHO/TDR 2009). Dengue fever is an ancient and well adapted human acute viral fever, causing mild symptoms in most cases. However, about ten percent patients evolve to severity, and the infection is currently considered as the most important emergent and re-emergent disease in respect to morbidity and mortality. In Brazil, it is placed among one of the most serious public health issues, and it had been reported about 1.5 million cases until the 37th week of 2016 (MS 2017).

In severe cases, vascular permeability leading to hypotension may evolve to shock and hemorrhagic manifestations may occur as well (WHO/TDR 2009). It is well known that a cytokine storm occurs because of immunological activation after DENV infection, exerting a crucial effect in the endothelial permeability (Srikiatkhachorn 2009).

Alterations in hepatic functionality are commonly described during dengue fever, including the increase in plasma aminotransferases and higher cytokine/chemokine circulation may be associated with liver dysfunctions in acute dengue (Ferreira et al. 2015). Hepatocytes are considered one of the DENV targets for infection (Limonta et al. 2007) and hepatocyte-derived cell lines have been used as targets for *in vitro* infection models (Chuang et al. 2011).

The species *Uncaria tomentosa* and *U. guianensis* (Rubiaceae) known as cat’s claw, unha-de-gato (Brazil) or uña-de-gato (Spanish America) are large woody vines, distributed from Central to South America, that have been used in traditional medicine largely in similar ways to treat several inflammatory and degenerative based illnesses, such as arthritis, diabetes, cancer, gastric ulcers, fevers and hemorrhages (Jones 1995, Zevallos-Pollito & Tomazello 2006).

In previous studies we have reported the antiviral and immunomodulatory activities of *U. tomentosa* on human monocytes infected with DENV-2 (Reis et al. 2008). Here, by adopting an *in vitro* DENV infection model with the hepatocyte cell line Huh-7 as target cell, we determined the inhibitory effects of hydro-alcoholic crude extracts from leaves and bark of *U. guianensis* assessed by virus load and inflammatory cytokine/chemokine production.

## MATERIALS AND METHODS

*Plant material, extraction and extract fingerprints* - *U. guianensis* was collected from wild in Rio Branco, Acre state, Brazil. The material was identified by the botanist Mario Gomes and a voucher specimen was deposited at the Herbarium of the Instituto de Biologia of the Universidade Federal do Rio de Janeiro, Brazil, under number RFA 36973.

Dried and sieved leaves (787 g) and bark (528 g) were extracted at room temperature with ethanol/water 1:1 v/v. The solvents were removed under low pressure yielding 187 g and 18 g of the dried extracts respectively.

The thin layer chromatography (TLC) analyses were performed in pre-coated silica gel 60 F254 (Merck) plates using: (a) mobile phase hexane/ethyl acetate 5:95 v/v and UV irradiation at 254 nm followed by spraying with Dragendorff reagent/sodium nitrite 10% to visualise spots, for alkaloid profiles (Valente et al. 2006); and (b) mobile phase ethyl acetate/formic acid/acetic acid/water 100:11:11:27 v/v and, to visualise spots: (b.1) UV irradiation at 365 nm, (b.2.) spraying with NP reagent and sequential UV irradiation at 365 nm and (b.3) spraying with iron chloride 10% solution, for flavonoid profiles (Wagner & Bladt 2009). Aliquots of 10 mL of methanol solutions at concentrations of 50 mg/mL for the extracts and 5 mg/mL for the references were applied to the plates. The ^1^H nuclear magnetic resonance (NMR) spectra were recorded at 25ºC on a Varian VNMRS500 (500 MHz) spectrometer in 5 mm NMR tubes, 18.1 mg [*U. guianensis* hydro-alcoholic extracts from leaves (UGL)] and 18.7 mg [*U. guianensis* hydro-alcoholic extracts from bark (UGB)] in 0.6 mL of deuterated dimethyl sulfoxide (DMSO-d_6_, Tedia) and solvent as internal reference. The alkaloid-rich fraction (UTAF) used as reference for the alkaloid profiles was obtained by acid-base partition from an ethanol/water 1:1 v/v extract of *U. tomentosa* bark and sequentially authenticated as a mixture of the six pentacyclic oxindole alkaloids (POA) currently considered as the *U. tomentosa* marker (Reis et al. 2008). Catechin was purchased from Sigma-Aldrich and kaempferitrin was previously isolated from *U. guianensis* leaves (Valente et al. 2009).

*Virus strain and viral stock* - Dengue virus type 2 originated from strain Thailand/16681/1984 (Halstead & Marchette 2003) was used for virus stock preparation as described elsewhere (Torrentes-Carvalho et al. 2009). Briefly, *Aedes albopictus* cell clone C6/36 (CRL-1660, ATCC) were maintained at 28ºC in Dulbecco’s modified Eagle Medium (Gibco, Life Technologies) with sodium bicarbonate (Sigma-Aldrich) and supplemented with 5% fetal bovine serum (FCS; Gibco, Life Technologies), 1% penicillin-streptomycin-glutamine (Gibco, Life Technologies), 0.5% non-essential amino acids (Gibco, Life Technologies) and 10% tryptose phosphate broth (Sigma-Aldrich). C6/36 cell monolayers were infected with DENV-2 and cell culture supernatants were harvested eight days later when cytopathic effect was observed. A concentrated DENV-2 stock was obtained by ultracentrifugation at 100,000 g for 1 h and set to a final volume 20 times smaller than initial (Gandini et al. 2013). Titration was performed in C6/36 cells using a standard TCID_50_ (50% tissue culture infective dose) assay as described elsewhere (Torrentes-Carvalho et al. 2009). Uninfected flasks were maintained, also concentrated and used as negative control (MOCK). Infectivity of ultracentrifuged virus inoculum was comparable with the original C6/36 supernatant regarding to concentrated volumes. It presented a titer of TCID_50_ = 5 x 10^7^/mL and was used at 1/100 dilution in Huh-7 assays. For control assays, the DENV-2 was inactivated by incubating the inoculum for 30 min/56ºC.

*Huh-7 cell line* - Human hepatocellular carcinoma cell line (Huh-7) were cultured in Dulbecco’s complete medium and supplemented with 10% FCS, 2 mM de L-glutamine, 100 µg/mL streptomycin and 100 U/mL de penicillin, all from Gibco, Life Technologies. Cells were maintained at 37ºC with 5% CO_2_ until 80% confluence.

*Cytotoxicity assay by MTT* - UGB and UGL stock solutions were prepared in 1% de DMSO and PBS pH 7.2-7.4 and gamma irradiated 200 rads for five hours.

Huh-7 cells were plated in 96-well flat microtiter plates (10^5^ cells/well) with Dulbecco’s complete and supplemented medium with 10% FBS until 80% confluence. Cells were further incubated with serial dilutions from UGB and UGL extracts. For performing cytotoxicity assay cells medium was replaced by RPMi 1640 medium without phenol red (Gibco, Life Technologies) and 10% MTT reagent (Vybrant® MTT Cell Proliferation Assay Kit, Life Technologies) and incubated 4 h at 37ºC with 5% CO_2_. Optical densities (OD) were determined at SpectraMax, Molecular Devices with Paradigm Software SoftMax® Pro 6, at 620 - 570 nm.

*Huh-7 infection with DENV-2 and UGB and UGL treatment* - Huh-7 cells were plated in 96-well flat microtiter plates (10^5^ cells/well) with Dulbecco’s complete and supplemented medium without FBS and viral inoculum was added at dilution 1/100. After the 2 h absorption period at 37ºC with 5% CO_2_ supernatants were replaced by serial dilutions of UGB or UGL in 2% FBS. Dexamethasone (0.1 mM) was used as positive control for antiviral activity (Reis et al. 2007). After the incubation period supernatants were collected and stored at -70º C. For flow cytometry assays cells were detached with trypsin-EDTA 0.25% (Life Technologies Cat#25200-56).

*Flow cytometry analysis for DENV antigen (Ag) detection* - Huh-7 cells that were detached after DENV-2 infection and UG treatment were centrifuged at 350 g/6 min/RT. Before antigen labeling, cells were permeabilised with 0.15% saponin (Sigma-Aldrich) 1% BSA, 0.1% azide in PBS pH 7.2-7.4 10 min/4ºC. Cells were washed incubated with blocking solution (1% bovine serum albumin/BSA, 0.1% azide and 5% human inactivated plasma in PBS) for 30 min/4ºC. After washing, cells were labeled with mouse monoclonal antibody anti-Dengue Complex (1:100 dilutions, Millipore cat # MAB8705, clone D3-2H2-9-21) (Puttikhunt et al. 2008) or isotype (1:100 dilution, cat# MABC004) and anti-mouse IgG Alexa Fluor® 488 (1:400 dilution, Life Technologies) for 60 min/4ºC. Cells were washed with 0.15% saponin, fixed with 2% paraformaldehyde (Sigma) and 2% FBS for 15 min/RT, washed and re-suspended in PBS. Cells were analysed by C6 Cytometer (Accuri/BD Biosciences). FlowJo software version X (Treestar) was used to analyse flow cytometry data.

*Cellular viability by Live/Dead® assay during UG treatment* - Huh-7 cells that were detached after DENV-2 infection and UG treatment were centrifuged at 350 g/6 min/RT and washed with PBS pH 7.2-7.4. Cell viability was detected by LIVE/DEAD*®* Fixable Read Dead Cell Stain Kits (Molecular Probes cat # L10120), according to the manufacturer’s protocol. Briefly, 1 x 10^6^ cells were incubated with the LIVE/DEAD*®* reagent for 30 min/4ºC. After washing the cells, DENV Ag was detected by intracellular immunofluorescence and flow cytometry analysis as detailed in the previous item.

*Detection of NS1 nonstructural DENV protein by enzyme-linked immunosorbent assay (ELISA)* - Supernatants from DENV infected and UG treated cells were thawed and NS1 detection was performed by Dengue NS1 Ag kit Platelia (BioRad). Samples were diluted 1:50. Plates were read at SpectraMax Paradigm Software SoftMax® Pro 6 at 450 nm.

*Detection of interleukin-6 (IL-6), interleukin-8 (IL-8) and macrophage migration inhibitory factor (MIF)* - Supernatants from DENV infected and UG cells were thawed and IL-6, IL-8 and MIF were detected by the following ELISA kits: Human IL-6 Matched Antibody Pairs (eBioscience); Human IL-8 ELISA Development Kit (PeproTech); Human MIF ELISA Development Kit (R&D Systems); according to the manufacturer’s instructions and using Streptavidin-HRP - (R&D Systems) diluted 1/200 in 1/5000 assay buffer (BSA 5%, Tween 20 0.5% in PBS 7.2-7.4), TMB (Sigma) and stop solution (H_2_SO_4_ 2N).

*Ethical and biodiversity rights* - The study was performed according to the international, national and institutional rules considering *in vitro* experiments and biodiversity rights. Genetic biodiversity property was authorised under n# 010579/2013-3 by Brazilian Council for Scientific and Technological Development (CNPQ) and under CAAE 13318113.7.0000.5248 by Human Ethical Comitee - Plataforma Brasil, FIOCRUZ.

## RESULTS

*Chemical profiles of the extracts* - Previous chemical studies of *U. guianensis* and *U. tomentosa* revealed for both species the presence of indole and oxindole alkaloids, proanthocyanidins, flavonols, triterpenoid glycosides and steroids (Heitzman et al. 2005, Valente et al. 2009). These compounds have distinct chemical characteristics: the indole and oxindole alkaloids are basic, with medium polarity; the flavonoids/flavonoid glycosides/proanthocyanidins have acidic characteristics and are considered polar compounds; the triterpene glycosides (polar compounds) and steroids (low-polar compounds) are neutral. This diversity in the chemical profile turns the access the whole metabolic fingerprint into a challenge.

**Fig. 1:**
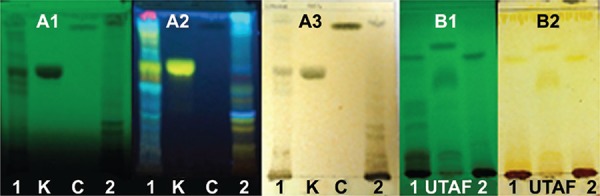
: thin layer chromatography profiles of the *Uncaria guianensis* extracts. 1: leaf extract; 2: bark extract. The A plates represent flavonoid profiles: mobile, phase ethyl acetate/formic acid/acetic acid/water 100:11:11:27; A1 under UV light at 254 nm; A2 spraying with NP reagent; A3 spraying with iron chloride solution; K = kaempferitin; C = catechin. The B plate represents alkaloid profiles: mobile phase, hexane/ethyl acetate 5:95; under UV light at 254 nm; UTAF = pentacyclic oxindole alkaloid enriched-fraction.

**Fig. 2:**
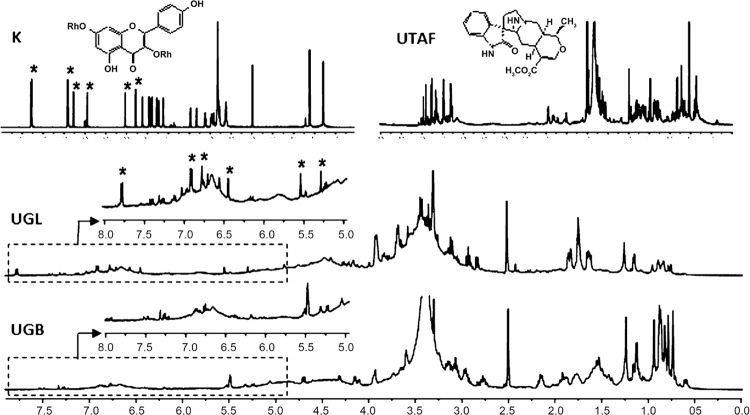
: 1H nuclear magnetic resonance (NMR) (400 MHz, DMSO-d6) profiles of the *Uncaria guianensis* extracts: UGL = leaf extract; UGB = bark extract; K = kaempferitrin; UTAF = pentacyclic oxindole alkaloid enriched-fraction.

To compare the secondary metabolite fingerprints of the studied extracts from *U. guianensis*, their chemical profiles in TLC and ^1^H NMR were accessed. TLC has been widely used as a primary prospecting fingerprint technique and ^1^H NMR has been increasingly employed as a rapid technique for the profiling of medicinal plants and has been able to detect a broad range of metabolites in a non-targeted way. The flavonoids kaempferitrin and catechin and a POA-rich fraction (Reis et al. 2008) were used as reference in both techniques. The results are shown in Figs 1 and 2, revealing some differences between the extracts. The TLC chromatograms (Fig. 1) indicated that both extracts contain the UG/UT alkaloid type profiles presenting characteristic spots with absorption under UV at 254 nm and after derivatisation with Dragendorff reagent (Valente et al. 2006) (Fig. 1 B1-B2). They also indicated that samples contain flavonoids/phenolics by presenting spots with bright green, blue and yellow colors after derivatisation with NP reagent and some with brown color after spraying with iron chloride solution. The leaf extract strongly suggested the presence of kaempferitrin (Fig. 1 A1-A3).

The ^1^H NMR spectra of the extracts, obtained in the same concentration and expanded on the same scale (Fig. 2 - UGL and UGB spectra), revealed low intensity signals in the aromatic proton region (d 6.5-8.0 ppm) and high intensity signals in the carbinolic proton region (sugar moiety) (d 3.0-4.0 ppm). The aliphatic proton region (d 0.5-1.5 ppm) showed some differences in the intensity of the signals. The low intensity of the majority aromatic signals in both extracts can be related to their possible low content of alkaloids that has been reported for *U. guianensis* (Sandoval et al. 2002). In the other hand, a comparison between the δ 5.0-8.0 ppm region of the kaempferitrin (K) spectrum with those of both studied extracts, showed only for the leaf extract detached signals at δ 7.78 (d, *J* = 8.7 Hz), 6.91 (d, *J* = 8.7 Hz), 6.78 (d, *J* = 1.8 Hz), 6.45 (d, *J* = 1.8 Hz), 5.54 (brs) and 5.29 (brs) such as found in the kaempferitrin spectrum, which correspond to the kaempferitrin aglycone protons and to its two anomeric α-ramnose protons, therefore confirming the presence of kaempferitrin in this extract. The data corroborated those previously found in other *U. guianensis* leaf extracts (Valente et al. 2009).

*Cytotoxicity of UGB and UGL extracts during Huh-7 hepatocyte cell line cultures using cell viability assay - MTT* - Both UGB and UGL extracts were tested by treating Huh-7 cells in culture up to 96 h at different concentrations. Cytotoxicity was evaluated according to OD obtained during the MTT assay. Untreated Huh-7 cells were used to normalise viability percentages and extract concentrations lower that 90% viability were considered toxic. UGB presented cytotoxic properties at concentration of 100 µg/mL even at 24 h after the treatment and UGL of 25 µg/mL and higher (Table I). Good viability was obtained with lower concentrations until 72 h, and all other assays were performed with nontoxic concentrations for three days and with daily measurements. No proliferative effect from UGB and UGL was detected, since all viability percentages were 100%, except for UGB at 1 and 10 µg/mL that presented 102% and 106%, that were not significantly elevated, reflecting slight variations in biological experimentation.

**TABLE I:** : *Uncaria guianensis* bark extract (UGB) and *U. guianensis* leaf extract (UGL) cytotoxicity in uninfected Huh-7 cells. Viability detection by MTT assay and comparison to control cells cultured without UG

UGB (µg/mL)					
Time after infection (h)	100	50	25	10	1
24	78*	97	98	100	99
48	87	93	98	102	106
72	83	94	96	98	99
96	76	79	76	78	84

UGL (µg/mL)					

Time after infection (h)	100	50	25	10	1

24	69	73	79	93	93
48	72	77	78	90	92
72	55	70	78	86	93
96	46	60	64	66	67

*: cellular viability rates determined after Huh-7 cell line cultures were treated or not with UGB or UGL for 24-96 h. Percentages were normalised according to control optical density in MTT assay (GraphPad Prism 6) and means of replicates are represented. Four independent experiments were tested with 3-8 replicates each.

*Live Huh-7 cell counts and live infected cells detected by Live/Dead® assay are changed after UGB and UGL treatments* - The loss of living cells after DENV-2 infection treated or not with UGB and UGL was analysed by flow cytometry after performing the Live/Dead® Assay. Absolute cell numbers were quantified and the percentages of these cells that were positive for DENV antigen (DENV-Ag^+^) were determined.

**Fig. 3:**
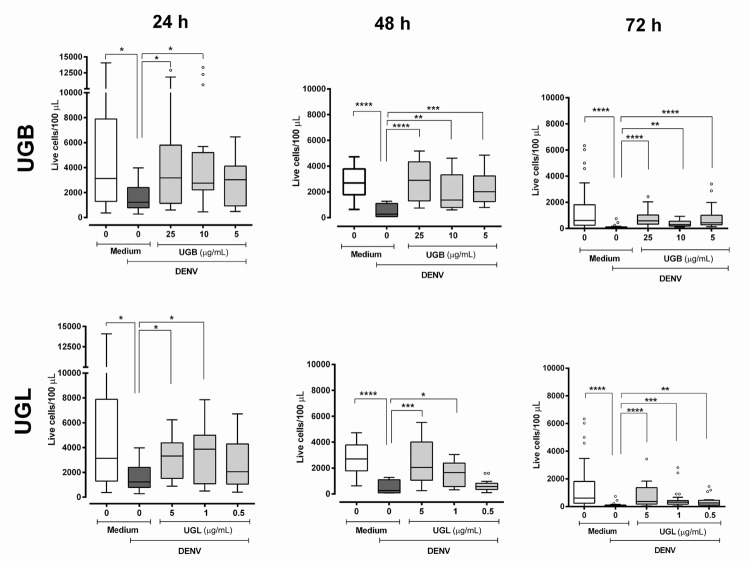
: LIVE/DEAD® viability of dengue virus (DENV)-2 infected Huh-7 cells treated with *Uncaria guianensis* bark (UGB) or leaf (UGL) extracts. Huh-7 hepatocyte cell line was infected with DENV-2 (TCID50 = 5 x 107/mL) for 24-72 h and viability was detected by the LIVE/DEAD® Fixable Dead Cell Stain Kit. Fluorescence intensity and percentage of labelled cells were determined using flow cytometry analysis (FL-4). Absolute cell counts were corrected according to the volume analysed. Six independent experiments with 3-4 replicates each are represented and were used for Kruskal-Wallis and Dunn’s multiple comparison tests. Significances: * p < 0.05, ** p < 0.01, *** p < 0.001, **** p < 0.0001.

During the three-day Huh-7 cell culture there was decay in absolute numbers of untreated living cell, but numerous cells remain viable in culture. The DENV infection reduces significantly the number of viable cells as compared to controls during the evaluation period (Fig. 3). Both UGB and UGL treatments associated to the DENV-2 infection are capable to maintain cell numbers significantly higher than those of untreated DENV infected cells in almost all concentrations tested during the three-day cultures, with a more marked effect at later periods of infection. At the concentration of 5 µg/mL used both extracts - UGB and UGL - were significantly effective, but no statistical differences were found for the number of cells between these two treatments. Moreover, heat-inactivated DENV or UGB/UGL extracts used in absence of virus did not alter significantly the counts of viable Huh-7 cells (Kruskal-Wallis with Dunn’s multiple comparisons test; Supplementary data, Fig. 1).

**Fig. 4:**
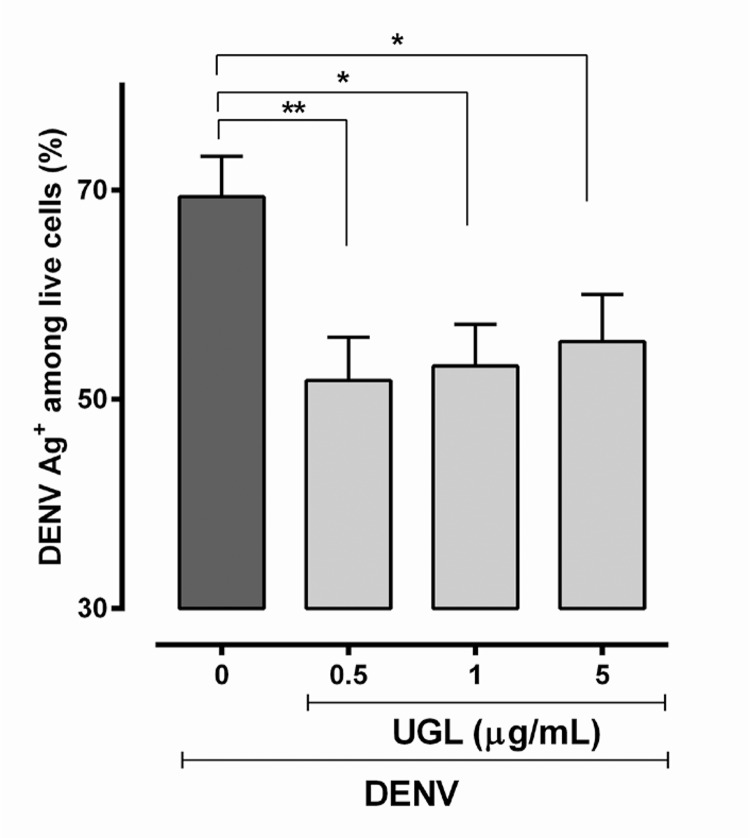
: dengue virus (DENV) antigens detected in live Huh-7 cell cultures after treatment with *Uncaria guianensis* bark (UGB) or leaf (UGL) extracts. Huh-7 hepatocyte cell line was infected with DENV-2 (TCID50 = 5 x 107/mL) for 24 h and treated with different concentrations of UGB, UGL or untreated. Viability was detected at 24 h by the LIVE/DEAD® Fixable Dead Cell Stain Kit. DENV antigen was detected by intracellular labeling with anti-DENV monoclonal antibody and anti-IgG (Alexa® 488). Fluorescence intensity and percentage of labelled cells were determined using flow cytometry analysis (FL-1 and FL-4). Six independent experiments with 3-4 replicates each are represented and were analysed by One way-ANOVA and Holm-Sidak’s multiple comparison tests. * p < 0.05 and ** p < 0.01.

It was possible to detect DENV antigens by a pre-M protein reactive antibody intracellularly (see M&M) in living cells by flow cytometry analysis at 24 hour-incubation (Fig. 4, Supplementary data, Fig. 2); however, the number of living cells was too small to allow further analyses at longer periods. We observed that only UGL was significantly decreasing the DENV-Ag^+^ cell rates at early infection. Assay control of heat-inactivated DENV presented very low frequencies of positive cells [mean ± standard error (SE)] = 1.59 ± 0.39 %, N = 6).

**Fig. 5:**
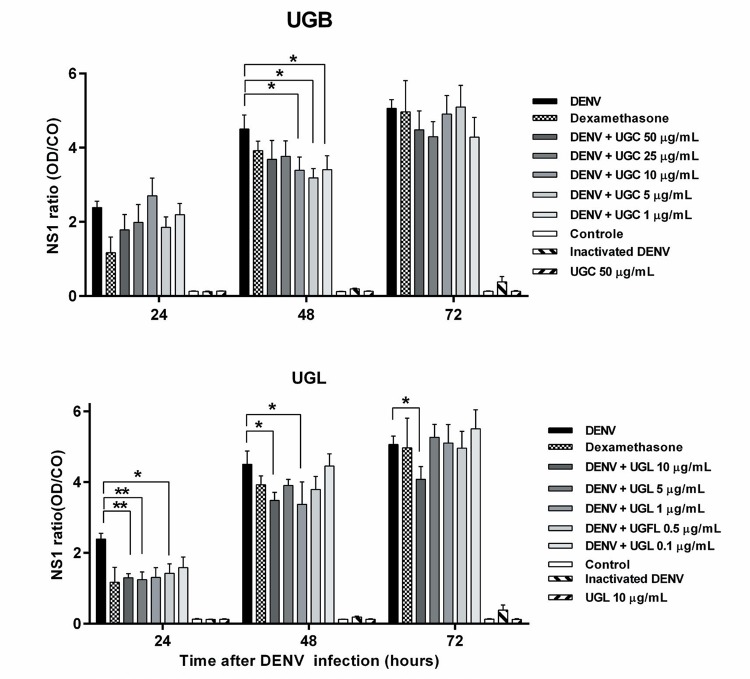
: non-structural protein NS1 detected in Huh-7 cell cultures after treatment with *Uncaria guianensis* bark (UGB) or leaf (UGL) extracts. Huh-7 hepatocyte cell line was infected with dengue virus (DENV)-2 (TCID50 = 5 x 107/mL) for 24-72 h and treated with different concentrations of UGB or UGL, dexamethasone (0.1 mM) or untreated. Non-structural protein NS1 was detected by enzyme-linked immunosorbent assay (ELISA) Dengue NS1 Ag kit Platelia in cell culture supernatants 24, 48 or 72 h after infection and treated UGL. The ratio represented was calculated the rate of the optical density (OD) obtained from samples and OD from NS 1 cut-off (average of duplicates from kit calibrator, CO). The values were represented as mean ± SEM. Three independent experiments with 1-3 replicates each are represented and were used for the Multiple *t*-test. * p < 0.05.

*UGB and UGL antiviral effect in NS1 secretion and intracellular DENV Ag detection in Huh-7 infected cells* - NS1 non-structural protein is secreted in the Huh-7 hepatocyte culture supernatants and their levels are increasing during the course of DENV-2 infection (Fig. 5). The NS1 protein detection was significantly decreased with UGL treatment markedly at 24 h at concentrations of 0.5, 5 and 10 µg/mL. At 48 h both preparations were efficient in diminishing NS1 production at concentration 1-10 µg/mL and at 72 h the concentration of 10 µg/mL UGL in cultures still presented mild by significant lower NS1 levels. Dexamethasone settled as inhibitory control had a borderline effect at 24 h (p = 0.05167). No differences in NS1 levels were detected at 5 µg/mL concentrations when UGB and UGL are compared.

**Fig. 6:**
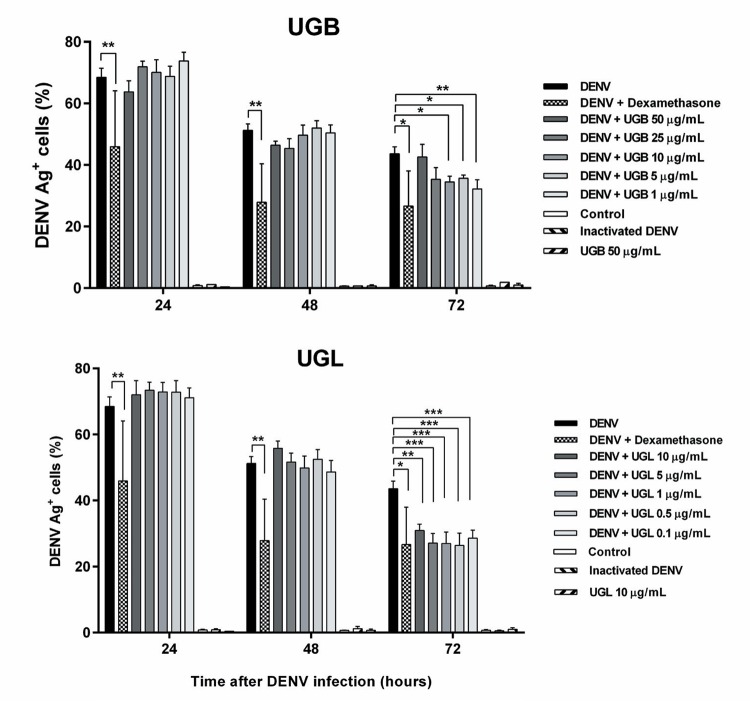
: total dengue virus (DENV) Ag+ cells detected in Huh-7 cell line after *Uncaria guianensis* treatment. Huh-7 hepatocyte cell line was infected with DENV-2 (TCID50 = 5 x 107/mL) for 24, 48 or 72 h and treated with different concentrations of *U. guianensis* bark extract (UGB), *U. guianensis* leaf extract (UGL) or untreated. DENV antigen (Ag) was detected by intracellular labeling with anti-DENV monoclonal antibody and anti-IgG (Alexa Fluor® 488). Fluorescence intensity and percentage of total labelled cells (live + dead) were determined using flow cytometry analysis (FL-1). Three independent experiments with 2-4 replicates each are represented and were analysed by one way-ANOVA and Holm-Sidak’s multiple comparison tests. * p < 0.05, ** p < 0.01 and *** p < 0.001.

We detected the intracellular DENV-Ag^+^ cell rates by flow cytometry analysis in regions containing cells with the Huh-7 morphology (FCS *vs.* SCC plots; Supplementary data, Fig. 3) and did not discriminate live from dead cells. DENV Ag was present at high frequencies at 24 h decreasing afterwards [mean ± standard error of the mean (SEM)]: 24 h = 68 ± 3, n = 24; 48 h = 51 ± 2, n = 21; 72 h = 44 ± 2%, n = 8 assayed in Huh-7 infected cells). During the first 48 h no differences in this detection of DENV Ag in total Huh-7 cells were statistically different after UG treatments, despite of dexamethasone presented a significant decrease in intracellular DENV Ag detection. However, at 72 h not only dexamethasone but also both UGB and UGL extracts were exerting an inhibitory effect in DENV-Ag^+^ cell rates, with a more pronounced effect with UGL at tested concentrations (Fig. 6, Supplementary data, Fig. 3). Treatments with the UGL extract at 5 µg/mL showed a significant decrease of DENV-Ag^+^ cell rates at 72 h as compared to UGB (mean ± SEM: UGB = 36 ± 1 % *vs*. UGL = 27 ± 3 % DENV-Ag^+^ cells with n = 12 in Multiple *t*-test comparison).

In general, UGL has more efficient effects in diminishing virus load either by the intracellular detection of pre-M antigen or by the NS1 secretion.

**Fig. 7:**
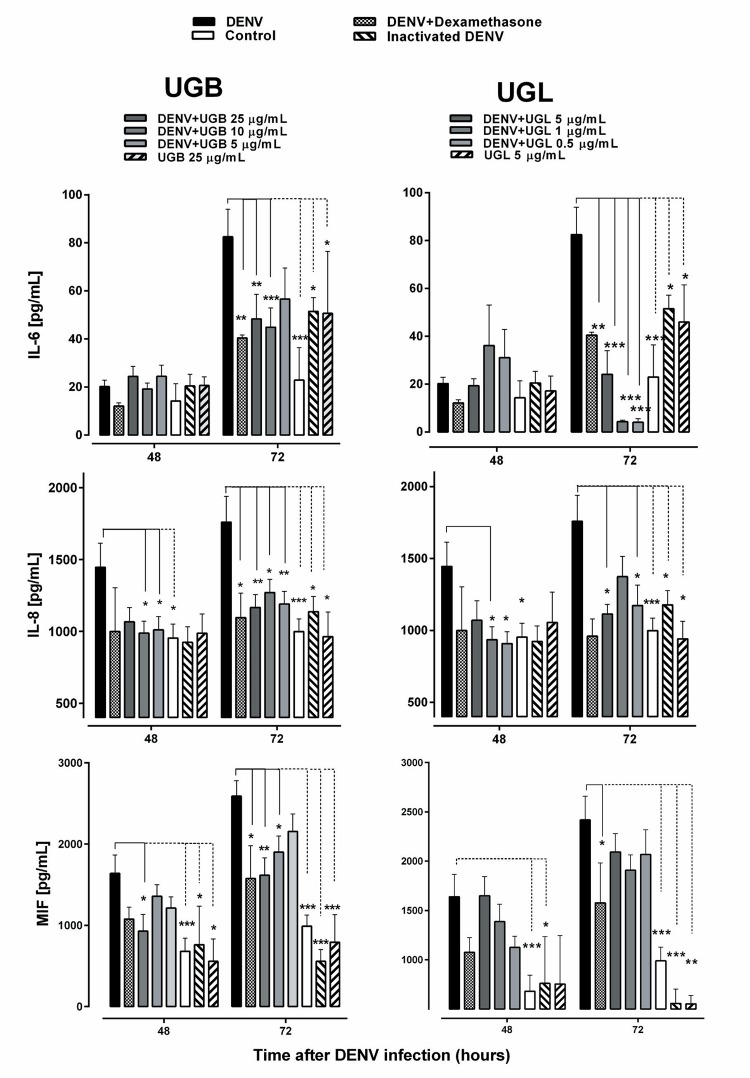
: cytokine/chemokine production by dengue virus (DENV)-2-infected Huh-7 cells treated with *Uncaria guianensis* bark extract (UGB) or *U. guianensis* leaf extract (UGL) for 48-72 h. Huh-7 hepatocyte cell line was infected with DENV-2 (TCID50 = 5 x 107/mL) for 48-72 h and treated with different concentrations of UGB, UGL or untreated. IL-6, IL-8 or macrophage migration inhibitory factor (MIF) were detected in cell culture supernatants by enzyme-linked immunosorbent assay (ELISA). Heat inactivated DENV was used as control. The values were represented as mean ± standard error mean (SEM). Three independent experiments with 1-3 replicates each are represented and were used for the Multiple *t*-test comparisons. * p < 0.05, ** p < 0.01.

*Cytokine/chemokine modulation by UGB and UGL* - Dengue patients characteristically display a cytokine storm during acute phase that result in severe pathophysiology such as vascular leakage (Srikiatkhachorn 2009). *In vitro* infected cells and specifically Huh-7 hepatocytes can produce cytokine/chemokines after DENV infection (Chuang et al. 2011). We evaluated some of those that are known as inducers of endothelial permeability in *in vitro* models. Interleukin-6 (IL-6), interleukin-8 (IL-8) and macrophage (Srikiatkhachorn 2009) migration inhibitory factor (MIF) were produced during Huh-7 DENV infection at significant levels in cell supernatants starting from 48 h after infection (Fig. 7). UGB inhibited significantly MIF and IL-8 levels from 48 to 72 h after infection and UGL inhibited IL-6 (72 h) and IL-8 (48-72 h). No significant differences were detected when extracts were compared at 5 µg/mL, except for IL-6 that presented lower levels by UGL treatment (mean ± SEM: 24 ± 10 pg/mL; n = 6) as compared to UGB (57 ± 13 pg/mL; n = 9) significant in Multiple t test comparison.

*Association among live cells, DENV/NS1 antigen detection and cytokine/chemokine production* - To evaluate the association of target cell viability, virus load, and immunological factors, we performed a series of correlation tests. Considering that various parameters tested in this work were not consistently from the same experiment, we used the average of values from the same treatment in each point and applied the Spearman’s correlation test (Table II).

**TABLE II:** : Correlation among live cells, dengue virus (DENV)/NS1 antigen detection and cytokine/chemokine production*a*

		Live cells^b^	NS1^c^	DENV^d^	IL-6^e^	IL-8	MIF
Live	r	t	-0.8091	0.7844	-0.4767	-0.5481	-0.7597
cells	p		< 0.0001	< 0.0001	0.0336	0.0101	< 0.0001
NS1	r			-0.9156	0.4361	0.5584	0.8403
	p			< 0.0001	0.0546	0.0085	< 0.0001
DENV	r				-0,4	-0,4909	-0.7792
	p				0.0806	0.0238	< 0.0001

a: average of values from the same treatment in each point were submitted to the Spearman’s correlation test. We included samples from DENV infected Huh-7 treated with *Uncaria guianensis* bark extract (UGB) at concentrations 25, 10 or 5 µg/mL or *U. guianensis* leaf extract (UGL) treated with 5, 1 or 0.5 µg/mL at time-points of 24, 48 or 72 h; b: live cell counts per 100 µL; c: NS1 ratio; d: percent of DENV +positive cells; e: IL-6, IL-8 and macrophage migration inhibitory factor (MIF) pg/mL.

**Fig. 8:**
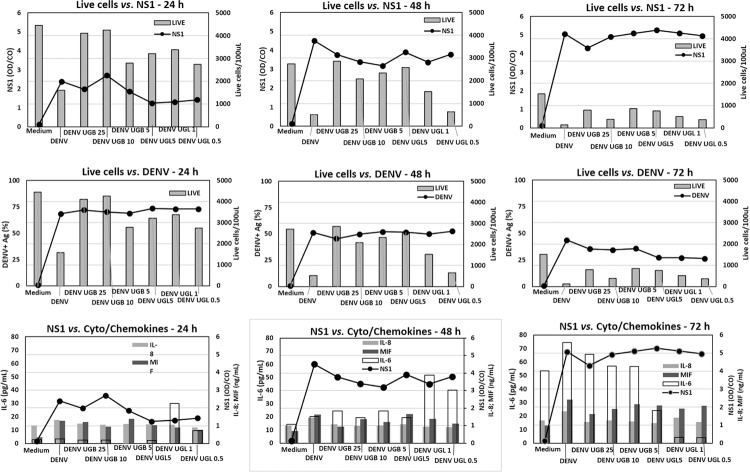
: association among live cells, dengue virus (DENV)/NS1 antigen detection and cytokine/chemokine production. Huh-7 hepatocyte cell line was infected with DENV-2 (TCID50 = 5 x 107/mL) for 24-72 h and treated with different concentrations of *Uncaria guianensis* bark extract (UGB), *U. guianensis* leaf extract (UGL) or untreated. We included samples from DENV infected Huh-7 treated with UGB at concentrations 25, 10 or 5 µg/mL or UGL treated with 5, 1 or 0.5 µg/mL at time-points of 24, 48 or 72 h. Live cell counts per 100 µL; NS1 ratio; percent of DENV positive cells; IL-6, IL-8 and macrophage migration inhibitory factor (MIF) = pg/mL.

The live cell counts were inversely correlated with NS1, IL-6, IL-8 and MIF. The less live cells were detected, the more these factors were produced. Intracellular DENV antigen was correlated with live cells and inversely correlated with NS1, IL-8 and MIF. NS1 was correlated with IL-8 and MIF. This is in accord to the fact that both live cells and intracellular DENV antigen decay with time. On the other hand, NS1 and IL-6, IL-8 and MIF increase with time. See Fig. 8.

## DISCUSSION

The induction of a hemodynamic deregulation and coagulation disorders are hallmarks for dengue fever. Moreover, liver dysfunctions such painful hepatomegaly, increase in aminotransferases are commonly present (Ferreira et al. 2015) and often associated with most severe cases. Several histopathological changes in liver have been reported, such as virus detection in hepatocytes (Limonta et al. 2007), the expression of inflammation related proteins such as Toll-like Receptors, inducible nitric oxide synthase and cytokines (IL-6 and IL-18) besides CD4 and CD8 T lymphocyte liver infiltration with the eventual presence of cytotoxic cell markers like granzyme B (Pagliari et al. 2014). The cytokine IL-6 and chemokines IL-8 and MIF are shown to be produced after the DENV challenge and represent factors that are associated with patient severity and induction of endothelial permeability (Srikiatkhachorn 2009, Ferreira et al. 2015).

Independently the success of DENV vaccines that are in advanced stages of development, specific therapeutic approaches not yet available to patients are certainly welcome. Considering the liver involvement in dengue pathophysiology, our infection model uses the Huh-7 hepatocellular carcinoma cell line as a virus target. It is known that hepatocytes are good targets for DENV and this cell line has been used to test antivirals against DENV (Cheung et al. 2014) and anti-inflammatory products (Li et al. 2012).

About one fourth of the commonly used medicines contain compounds isolated from plants and a variety of medicinal plants are reported to be used for treating viral infections (Mukhtar et al. 2008). The use of those having not only antiviral but also immunomodulatory properties are interesting approaches for developing therapies against viral infections.

UGB and UGL promoted protective effects in cell viability during DENV-infected Huh-7 cell line, indicating a possible beneficial effect on liver cells and occasionally in other tissues. We could speculate that an antiviral effect was achieved by both UG extracts, helping cell integrity maintenance. This effect was assayed either by detection of intracellular antigen detection or by NS1 accumulation in cell culture supernatant. Flow cytometry analysis for detecting intracellular DENV Ag is comparable to plaque forming assay to detect antiviral effects of test compounds (Fu et al. 2014) and NS1 was also used to test anti-DENV compounds presenting a slightly later peak than plaque forming assay (Schul et al. 2007). We cannot fully discard that particles released at Huh-7 culture, may have influence of a direct virucidal activity and this effect could contribute to the late antiviral activity evidenced at later time points.

Correlation tests lead us to hypothesise that effects of UG extracts on target cell viability and NS1 production are associated with cytokine production. As intracellular DENV Ag decays, extracellular NS1 elevates as a result of antigen accumulation. Antiviral effects from UGL were detected in living cells at 24 h labelling intracellular antigens. At this time-point it was possible to detect as well the NS1 downregulation in cell culture supernatants. At 72 h the effect on NS1 reduction may have been undermined by an excess of antigen and limitations in OD measured by the ELISA assay. Only a mild effect was detected by UGL at 10 µg/mL, despite of the marked reduction in intracellular antigens. Other techniques such as detection of viral nucleic acid may clarify this event. A moderate effect in DENV Ag decrease was also observed under UGB treatment, although significantly less than that observed by UGL at comparable doses.

Some reported investigations with *U. guianensis* have drawn our attention by displaying interesting anti-inflammatory effects in models using bacterial lipopolysaccharides, ovalbumin or zymosan (Carvalho et al. 2006). They have shown the plant inhibiting effect on ciclooxigenase-2 (COX-2), nitric oxide synthase, IL-6, TNF-α, p65 nuclear factor-κB (NF-κB), nitric oxide and IL-8 production *in vitro* as well in experimental models by reduction of paw edema and pleural exudation. We evaluated the immunomodulatory potential of our extracts and observed downregulation of IL-6, IL-8 and MIF besides the antiviral effect. Very few investigations have been reported with respect to the anti-inflammatory or immunomodulatory properties of anti-dengue products. Besides our own work, some mouse studies were performed (Schul et al. 2007) showing the inhibitory effect for cytokine circulating levels.

Among immunological factors studied here, IL-6, IL-8 and MIF are well known to change endothelial permeability (Srikiatkhachorn 2009, Chuang et al. 2011) and can be associated with dengue fever severity. Only a few groups have been administrating immunomodulatory therapies for dengue patients. Results are not yet conclusive with respect to beneficial effects. Chloroquine has been primarily used as an antimalarial drug. Due its anti-inflammatory effects it has been used recently in Brazilian dengue patients, showing a substantial decrease in pain intensity and a great improvement regarding their ability to perform daily activities during the chloroquine use (Borges et al. 2013). Also, few clinical trials are reported with the use of natural products in dengue. A propolis extract used in a small clinical trial showed a faster recovery in platelet counts, greater decline in circulating TNF and shorter hospitalisation period compared with placebo treated patients (Soroy et al. 2014).

The importance of the traditional use of *U. tomentosa* and *U. guianensis* in inflammatory processes has motivated several studies that have confirmed both two species as active for several inflammation processes (Sandoval et al. 2002, Carvalho et al. 2006). The search for a relationship between the anti-inflammatory activity and active principles seem to indicate a synergistic action of different classes of compounds present in the species (Aquino et al. 1991, Heitzman et al. 2005) The flavonol glycoside kaempferitrin present only in UGL has been studied in other plants showing immunomodulatory and anti-inflammatory effects, such as pain and paw edema inhibition (de Melo et al. 2009). These evidences corroborate our hypothesis that kaempferitrin may play an important role in the UGL stronger antiviral and immunomodulatory effects observed during the DENV infection in hepatocytes as compared to UGB effects.

Based on the traditional use and on literature reports of *U. guianensis* anti-inflammatory effects, we assessed an *in vitro* DENV-2 infection model and used its leaf and bark hydro-ethanolic extracts to identify a decrease in virus load and in reduction of cytokine/chemokine production by for dengue infected cells. The present data contribute to expand the knowledge about this medicinal plant; however, further studies may be performed to consider these products as potential candidates for the development of a herbal product for the future treatment of dengue.

## References

[B1] Aquino R, Defeo V, Desimone F, Pizza C, Cirino G (1991). Plant metabolites - new compounds and antiinflammatory activity of Uncaria tomentosa. J Nat Prod.

[B2] Borges MC, Castro LA, Fonseca BAL da (2013). Chloroquine use improves dengue-related symptoms. Mem Inst Oswaldo Cruz.

[B3] Carvalho MV, Penido C, Siani AC, Valente LM, Henriques MG (2006). Investigations on the anti-inflammatory and anti-allergic activities of the leaves of Uncaria guianensis (Aublet) J. F. Gmelin. Inflammopharmacology.

[B4] Cheung YY, Chen KC, Chen H, Seng EK, Chu JJ (2014). Antiviral activity of lanatoside C against dengue virus infection. Antiviral Res.

[B5] Chuang YC, Lei HY, Liu HS, Lin YS, Fu TF, Yeh TM (2011). Macrophage migration inhibitory factor induced by dengue virus infection increases vascular permeability. Cytokine.

[B6] Melo GO de, Malvar DC, Vanderlinde FA, Rocha FF, Pires PA, Costa EA (2009). Antinociceptive and anti-inflammatory kaempferol glycosides from Sedum dendroideum. J Ethnopharmacol.

[B7] Ferreira RA, Oliveira SA de, Gandini M, Ferreira LC, Correa G, Abiraude FM (2015). Circulating cytokines and chemokines associated with plasma leakage and hepatic dysfunction in Brazilian children with dengue fever. Acta Trop.

[B8] Fu Y, Chen YL, Herve M, Gu F, Shi PY, Blasco F (2014). Development of a FACS-based assay for evaluating antiviral potency of compound in dengue infected peripheral blood mononuclear cells. J Virol Methods.

[B9] Gandini M, Gras C, Azeredo EL, Pinto LM, Smith N, Despres P (2013). Dengue virus activates membrane TRAIL relocalization and IFN-alpha production by human plasmacytoid dendritic cells in vitro and in vivo. PLoS Negl Trop Dis.

[B10] Halstead SB, Marchette NJ (2003). Biologic properties of dengue viruses following serial passage in primary dog kidney cells: studies at the University of Hawaii. Am J Trop Med Hyg.

[B11] Heitzman ME, Winiarz E, Vaisberg AJ, Hammond GB (2005). Ethnobotany, phytochemistry and pharmacology of Uncaria (Rubiaceae). Phytochemistry.

[B12] Jones K (1995). Cat’s claw, healing vine of Peru.

[B13] Li XH, McGrath KC, Nammi S, Heather AK, Roufogalis BD (2012). Attenuation of liver pro-inflammatory responses by Zingiber officinale via inhibition of NF-kappa B activation in high-fat diet-fed rats. Basic Clin Pharmacol Toxicol.

[B14] Limonta D, Capo V, Torres G, Perez AB, Guzman MG (2007). Apoptosis in tissues from fatal dengue shock syndrome. J Clin Virol.

[B15] MS - Ministério da Saúde Monitoramento dos casos de dengue, febre de chikungunya e febre pelo vírus Zika até a Semana Epidemiológica 51, 2016.

[B16] Mukhtar M, Arshad M, Ahmad M, Pomerantz RJ, Wigdahl B, Parveen Z (2008). Antiviral potentials of medicinal plants. Virus Res.

[B17] Pagliari C, Quaresma JA, Fernandes ER, Stegun FW, Brasil RA, Andrade HF de (2014). Immunopathogenesis of dengue hemorrhagic fever: contribution to the study of human liver lesions. J Med Virol.

[B18] Puttikhunt C, Keelapang P, Khemnu N, Sittisombut N, Kasinrerk W, Malasit P (2008). Novel anti-dengue monoclonal antibody recognizing conformational structure of the prM-E heterodimeric complex of dengue virus. J Med Virol.

[B19] Reis SR, Valente LM, Sampaio AL, Siani AC, Gandini M, Azeredo EL (2008). Immunomodulating and antiviral activities of Uncaria tomentosa on human monocytes infected with Dengue Virus-2. Int Immunopharmacol.

[B20] Reis SRNI, Sampaio ALF, Henriques MGM, Gandini M, Azeredo EL, Kubelka CF (2007). An in vitro model for dengue virus infection that exhibits human monocyte infection, multiple cytokine production and dexamethasone immunomodulation. Mem Inst Oswaldo Cruz.

[B21] Sandoval M, Okuhama NN, Zhang XJ, Condezo LA, Lao J, Angeles FM (2002). Anti-inflammatory and antioxidant activities of cat’s claw (Uncaria tomentosa and Uncaria guianensis) are independent of their alkaloid content. Phytomedicine.

[B22] Schul W, Liu W, Xu HY, Flamand M, Vasudevan SG (2007). A dengue fever viremia model in mice shows reduction in viral replication and suppression of the inflammatory response after treatment with antiviral drugs. J Infect Dis.

[B23] Soroy L, Bagus S, Yongkie IP, Djoko W (2014). The effect of a unique propolis compound (Propoelix) on clinical outcomes in patients with dengue hemorrhagic fever. Infect Drug Resist.

[B24] Srikiatkhachorn A (2009). Plasma leakage in dengue haemorrhagic fever. Thromb Haemost.

[B25] Torrentes-Carvalho A, Azeredo EL, Reis SRI, Miranda AS, Gandini M, Barbosa LS (2009). Dengue-2 infection and the induction of apoptosis in human primary monocytes. Mem Inst Oswaldo Cruz.

[B26] Valente LMM, Alves FF, Bezerra GM, Almeida MBS, Rosario SL, Mazzei JL (2006). Development and application of methodology by thin layer chromatography to determine the profile of pentacyclic oxindole alkaloids in the South American species of the genus Uncaria. Rev Bras Farmacogn.

[B27] Valente LMM, Bizarri CHB, Liechocki S, Barboza RS, Paixão D, Almeida MBS (2009). Kaempferitrin from Uncaria guianensis (Rubiaceae) and its potential as a chemical marker for the species. J Braz Chem Soc.

[B28] Wagner H, Bladt S (2009). Plant drug analysis: a thin layer chromatography atlas.

[B29] WHO/TDR - World Heath Organization/Special Programme for Research and Training in Tropical Diseases (2009). Dengue: guidelines for diagnosis, treatment, prevention and control.

[B30] Zevallos-Pollito PA, Tomazello M (2006). Wood anatomy of Uncaria guianensis and U. tomentosa (Rubiaceae) from the state of Acre, Brazil. Acta Amazon.

