# Supplementary material for: Decrease in Dengue virus-2 infection and reduction of cytokine/chemokine production by Uncaria guianensis in human hepatocyte cell line Huh-7
Source: Mem Inst Oswaldo Cruz. 2017 Jun;112(6):458–68. doi: 10.1590/0074-02760160323 (PMC5446237; doi:10.1590/0074-02760160323)
Supplement: Supplementary file 1 [file 0074-0276-mioc-112-6-0458-suppl01.pdf]

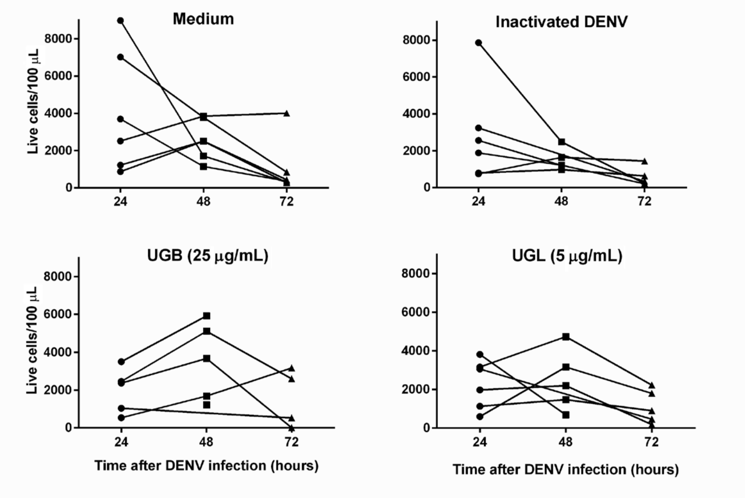

Fig. 1: LIVE/DEAD® viability of Huh-7 cells treated only with inactivated dengue virus (DENV)-2, *Uncaria guianensis* bark (UGB) or *U. guianensis* leaf (UGL) extracts for 24-72 hours. Huh-7 hepatocyte cell line was treated with culture medium, inactivated DENV-2, UGB (25 µg/mL) or UGL (5 µg/mL). Viability was detected by the LIVE/DEAD® Fixable Dead Cell Stain Kit. Fluorescence intensity and percentage of labelled cells were determined using flow cytometry analysis (FL-4). Absolute cell counts were corrected per the volume analysed. Numbers represent cell counts per 100 µL.

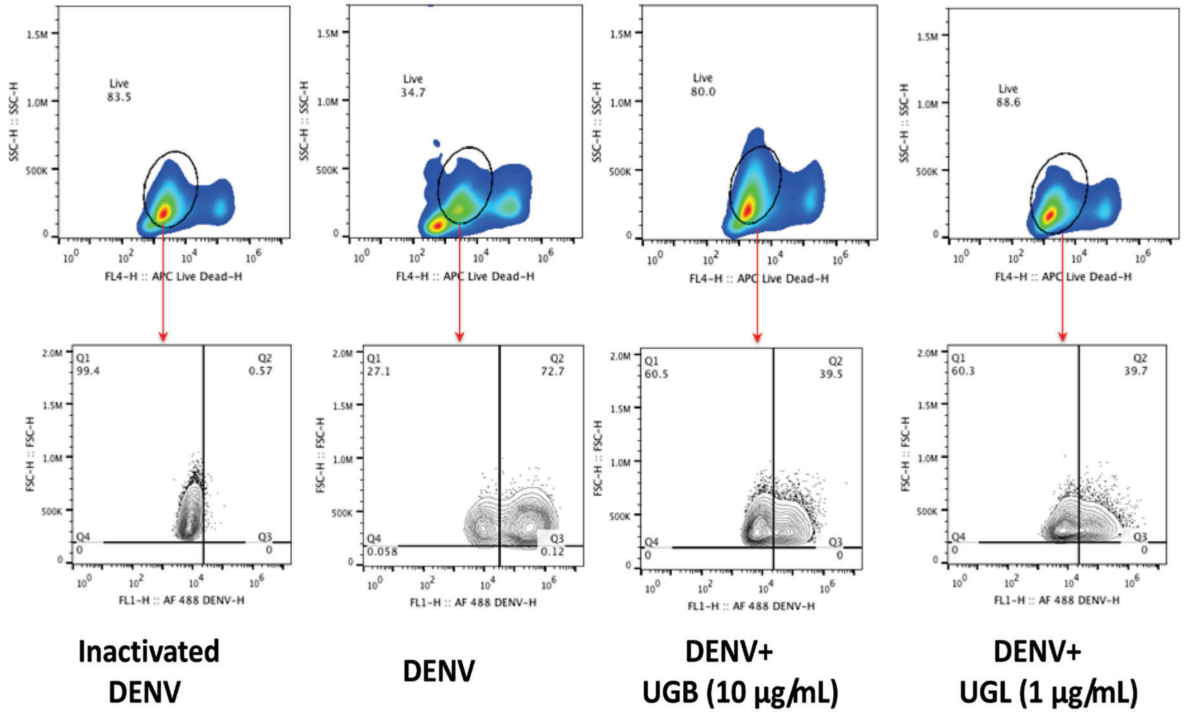

Fig. 2: Huh-7 hepatocyte profile for Live/Dead® labelling and dengue virus (DENV) detection in live cells. Huh-7 hepatocyte cell line was infected with DENV-2 ($TCID_{50}$ = 5 x 10^7/mL) for 24-72 h and treated with different concentrations of *Uncaria guianensis* bark (UGB), *U. guianensis* leaf (UGL) extracts or untreated. Viability was detected by the LIVE/DEAD® Fixable Dead Cell Stain Kit. DENV antigen was detected by intracellular labeling with anti-DENV monoclonal antibody and anti-IgG (Alexa Fluor® 488). Fluorescence intensity and percentage of labelled cells were determined using flow cytometry analysis (FL1 and FL4). After gating selection for doublet exclusion, living cells were selected and percentage of DENV antigen positive cells was determined. Huh-7 cultures incubated for 24 h with inactivated DENV, infectious DENV, DENV+UGB or DENV+UGL are represented.

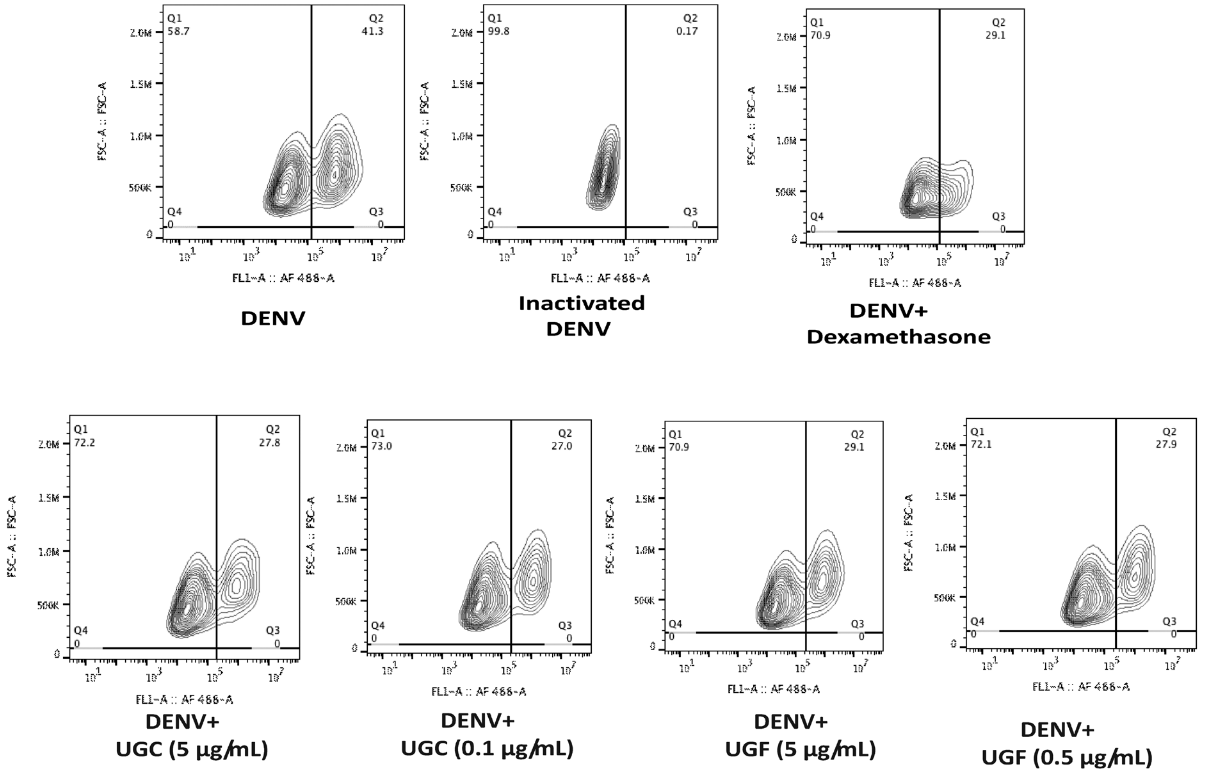

Fig. 3: dengue virus (DENV) detection in Huh-7 hepatocyte cells. Huh-7 hepatocyte cell line was infected with DENV-2 ($TCID_{50}$ = 5 x 10^7/mL) for 72 h and treated with different concentrations of *Uncaria guianensis* bark (UGB), *U. guianensis* leaf (UGL) extracts, dexamethasone or untreated. DENV antigen was detected by intracellular labeling with anti-DENV monoclonal antibody and anti-IgG (Alexa Fluor® 488). Fluorescence intensity and percentage of labelled cells were determined using flow cytometry analysis (FL1). After gating selection for doublet exclusion, percentage of DENV antigen positive living cells was determined. Huh-7 cultures incubated for 72 h with inactivated DENV-2, infectious DENV-2, inactivated DENV-2, DENV-2+Dexamethasone, DENV-2+UGB or DENV-2+UGL are represented.
